# Mr. Taylor, in Answer to Dr. Mossman

**Published:** 1809-08

**Authors:** Thomas Taylor

**Affiliations:** Harleston, Norfolk


### *To the Editors of the Medical and Phyfical Journal.*

GENTLEMEN

ACCORDING to Dr. Mossman's own shewing, I have neither misquoted the works to which he referred us, nor mistated the opinions, such as they are, of their authors. The Doctor, therefore, has only to complain, that I cited from those authorities the passages which were in my own favour. I am also obliged to him for extracting from Withering and Ferriar a page and a half of letter-press, which is to the full as much in support of my opinion as his own. On the subject of the digitalis then, he has generously lent his aid to prove, what I mainly contended for, viz. that the opinions of the writers to whom he appealed, were contradictory to each other, and that the estimate entertained of the virtues of this plant had considerably fallen in the minds of others.

In the first page of Doctor Mossman's paper, he has availed himself of the common notion respecting the importance of abating the irritating fever in phthisis by diminishing the velocity of the blood's current through the lungs; for that the principal wear and tear of the system is occasioned by this excited pulse. With this view, likewise, I believe, the physicians of the early part of the last century recommended the frequent use of the lancet in phthisical cases. I do conceive this view of the case, if not fundamentally erroneous, is at leat gratuitous; for I strongly suspect we are here mistaking the effect for the cause. The former is in the lungs. The accelerated pulse, the colliquative perspirations alternating with wasting diarrhœa, &c. are merely attendant upon and indicative of the magnitude of the evil. You may by bleeding, and still more effectually, perhaps, by the fox glove, lower the pulse and obscure the symptoms, but the mischief is advancing *viresque acquirit eundo.* To me it appears vastly more probable, that the great difficulty, I had almost said impossibility, impossibility, of curing by medicine an ulcer in the lungs, is the joint result of their peculiar structure and incessant movement in the business of respiration.

Doctor Mossman can have seen but little, or, which is more probable, much to very little purpose, if he have not frequently observed phthisical patients whose pulse has been reduced steadily below 80 for weeks together, by means of digitalis, gradually sinking under the irresistible progress of the disease, and whose sufferings were not only not alleviated by the influence of the fox glove, but the patients have *appeared* to gain renovated vigour, and called themselves better, on this medicine being withheld. This *apparent* alteration for the better, on the cessation of digitalis influence, is sometimes so obvious as to impress the friends of the patient with a belief, that an important and salutary change has taken place in the condition of the disorder, though at the same time, it is hurrying on to a fatal termination.

Hypothetical reasoning is probably on no subject more dark, fanciful, visionary, and inconsistent than on topics of disease. We talk of curing ulcers in the lungs by lessening the velocity of the blood's current through them. When we speak of the difficulty of healing ulcers in the legs, we then shift our ground, point our artillery in an opposite direction, and considering the too sluggish movement of the same fluid in the extremities as the chief obstacle to be combated, endeavour by every means, both within and without, to whip on the torpid stream.

I do not know any thing so calculated to bring discredit upon the profession and its professors, as the pomp with which medicines of doubtful or insignificant virtues are sent forth into the world. With all the boasted aid of the fox glove, what is the answer we obtain on examining the bills of mortality, when we ask whether the number of those who annually die of phthisis has regularly increased or decreased since the introduction of digitalis into common practice? Is it not a notorious and melancholy fact, that the number is greatly on the increase. And when it is asked by some, how was this medicine given, by whom, under what circumstances, and in what dose? This has too much the appearance of mystery for regular practice.

The wholesome maxim of antiquity, νῆφε καὶ μέμνασ' ἀπιστεῖν was early and carefully impressed upon my mind; and I must take the liberty of hinting, that it is never more necessary to be observed than when reading the lucubrations of medical writers.

When I find one practitioner extolling as a remedy in typhus fever, the bark and sulphuric acid as alone to be relied on;—another, cold water;—another, ardent spirit; another, to plunge the patient in a bath of animal broth;—another, mercurial unction: when one asserts, that the seat of this fever is fixed in the brain, and another, that it is in the stomach and bowels; I ask, what are we to infer from this mass of contradiction and wild conjectures? And whether it is unreasonable to exclaim, as Voltaire did at the incredible narrations of historians, *mais pour les miracles d' Esculape, n'en croyez rien!* So likewise, when I hear, in regard to the treatment of phthisis pulmonalis, one enjoining bleeding and a vegetable diet;—another, starvation and sleeping on a boarded floor;—another, a tonic plan ab initio;—another, steel and myrrh;—one to send phthisical patients to the pure air and sunny regions of Lisbon;—another, to the humid atmosphere and chilling fogs of Batavia;—and, to close the catalogue, when others talk of specifics for consumption, I again cry, *n'en croyez rien.*

Allow me, Gentlemen, on taking my FINAL LEAVE of Doctor Mossman, to tender you my thanks for the indulgence I have received from you.

I am, &c.

*Harleston, Norfolk.* THOMAS TAYLOR.

P. S. In imitation of Doctor Mossman's solicitude to bring me acquainted with books, I recommend to *his* ATTENTION a book published some twenty years ago, under the title of, The Medical Quixote. By the bye, so good a book deserves a much better title. T. T.

---

*⁂* As we inserted Dr. Mossman's last paper on condition that he would permit us to prune it, justice seemed to demand that we should do the same for Mr. Taylor. But from this time we must close the controversy. Communications of any other kind, from the same Gentlemen, shall meet with due attention.

