# Errata

**Published:** 1809-10

**Authors:** 


### ERRATA.

Page 124, line 8 from the bottom, for *former*, read *fomes*.

